# Assessing Whether Parents and Children Perceive the Meaning of the Items in the PedsQLTM 4.0 Quality of Life Instrument Consistently: A Differential Item Functioning Analysis

**DOI:** 10.5539/gjhs.v5n5p80

**Published:** 2013-06-06

**Authors:** Peyman Jafari, Zahra Bagheri, Seyyedeh Zahra Hashemi, Keivan Shalileh

**Affiliations:** 1 Department of Biostatistics, Shiraz University of Medical Sciences, Shiraz, Iran; 2 Tehran University of Medical Sciences, Tehran, Iran

**Keywords:** differential item functioning, children, parents, quality of life

## Abstract

Limited studies have examined the effect of differential item functioning (DIF) on comparing health related quality of life (HRQoL) scores across child self-reports and parent proxy-reports. This study aims to determine whether parents and children respond differently to the items in the Persian version of the PedsQoL™ 4.0 measure. The PedsQL™ 4.0 Generic Core Scales was completed by 938 child-parent dyads. The graded response model (GRM) was used to detect DIF between parents and children. The IRT analyses were conducted using IRTPRO 2.1. On the whole, our findings showed that 50% (4 out of 8) of the items in the physical subscale and 40% (2 out of 5) in both emotional and school subscales were flagged with DIF. Among the DIF items, 62.5% (5 out of 8) were uniform and the remaining 37.5% (3 out of 8) were non-uniform. Parents and children interpret certain items of the PedsQL™ 4.0 in a different ways, except for the social subscale. Hence, we should be cautious about using parent proxy-report as a substitute for a child's ratings.

## 1. Introduction

The issue of agreement between child self-reports and parent proxy-reports has been always a controversial aspect of measuring HRQoL in children and adolescents (Eiser & Morse, 2001; Upton, Lawford, & Eiser, 2008; Huang et al., 2009). As compared with adults, children may not understand the abstract concepts involved in health related quality of life (HRQoL) research. In the field of pediatrics, parent proxy-report can give valuable insight into the child's HRQoL, especially for children who are very young or otherwise unable to complete measures themselves (Vincent & Higginson, 2003). However, a question that arises is to what extent parent perception of their child's HRQoL can be a reliable substitute for the child self-report. Eiser and Morse's systematic review provides support for the view that agreement is higher between parents and chronically ill children than between parents and healthy children (Eiser et al., 2001). Moreover, in a more recent systematic review, Upton and colleagues identified that parents of healthy children tended to report better child HRQoL scores than children themselves, while parents of children with health conditions tended to underestimate child HRQoL (Upton et al., 2008). However, in all these studies, parent-child agreement has been evaluated at the scale level and not at the item level. Hence, these comparisons can be misleading because it is not clear whether the disparity in HRQoL is a real difference or it is a reflection of an artificial effect such as different interpretation of items by children and parents (Teresi & Fleishman, 2007).

Differential item functioning (DIF) is an efficient method to evaluate measurement equivalence between children and parents by assessing whether the probability of responding to an item between the groups is the same conditioning on the same level of the underlying HRQOL (Teresi, 2007). If the probability of endorsing an item is different across the groups, the comparisons between the scores of parents and children are meaningless. Two types of DIF, uniform and non-uniform, can be detected. Uniform DIF occurs when the difference in item response probabilities is constant across the scale. Non-uniform DIF is evident when the direction of DIF differs in different parts of the construct scale (Traebert et al., 2010; Traebert et al., 2010).

As far as we know, two studies have recently examined DIF between children and their parents through multiple-group confirmatory factor analysis (MCFA) in the PedsQL™4.0 instrument (Huang et al., 2009; Lin et al., 2012). Methodological experts believe that DIF analyses using the item response theory (IRT) model are more powerful than other existing DIF detection tests (Langer et al., 2008). The IRT model can be used to detect uniform and non-uniform DIF, can be used with items that have been polytomously scored, and have criteria available for estimation of the magnitude of DIF (DeMars, 2010; Embretson & Reise, 2000; Ostini & Nering, 2006). The aim of this study is to use the unidimensional IRT graded response model to determine whether parents and children perceive the meaning of the specific items in the PedsQL™ 4.0 consistently.

## 2. Methods

### 2.1 Study Population

The Persian version of the PedsQL™ 4.0, which had been translated and validated previously in Iran (Jafari, Ghanizadeh, Akhondzadeh, & Mohammadi, 2011; Jafari, Forouzandeh, Bagheri, Karamizadeh, & Shalileh, 2011), was completed by 938 school children (52.8% boys, 47.2% girls) and their parents in 80 classes (40 middle school classes and 40 high school classes) at 20 middle schools and 20 high schools (Jafari, Bagheri, Ayatollahi, & Soltani, 2012). The participants were randomly selected by a two-stage cluster random sampling technique from the four educational districts of Shiraz, southern Iran. Children aged 8 to 18 years, when child-parent dyads completed both child self-report and parent proxy-report versions, met our inclusion criteria to participate in the study. In addition, if more than 50% of the items in each self- and proxy-reports were missing, the dyads were not considered for analysis. As a result, we excluded approximately 312 subjects from the study. A trained researcher explained the survey to children in each classroom and distributed the informed consent forms and questionnaires for students to take home to their parents. Parents completed the questionnaire and returned them to school via students. The students completed the child self-reports at home after the parents gave informed consent; therefore, no child assent was sought. The study was approved by the ethical committee of our institution, Shiraz University of Medical Sciences. The consent rate in all classes was above 75%. The mean (± standard deviation) age of boys and girls was 14.36±2.20 and 13.88±2.41, respectively.

### 2.2 Instrument

The 23-item PedsQL™ 4.0 consists of four domains including physical health (8 items), emotional functioning (5 items), social functioning (5 items), and school functioning (5 items). The participants responded to the items on a 5-point Likert scale (0 = never a problem, 1 = almost never a problem, 2 = sometimes a problem, 3 = often a problem, and 4 = almost always a problem). According to the PedsQL™ 4.0 scoring algorithm, all rating scale categories of negatively worded items were reversed such that higher scores indicated better HRQoL, so models were fit accordingly.

### 2.3 Statistical Analysis

The GRM was used in this study for evaluating measurement equivalency (DIF) between children and their parents. The mathematical function for the GRM is


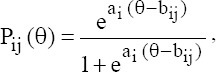


where P_ij_(θ) is the probability of scoring in or above category j of item i, a_i_ is the item discrimination (or slope) parameter, b_ij_ is the boundary location or threshold for category j of item i, and θ represents the continuous latent trait (person location). When higher scores correspond to greater quality of life, categories with larger b_ij_ parameters would be more likely to be endorsed by respondents with better quality of life than those with poorer quality of life.

In the GRM framework, two different types of DIF can be distinguished by comparing estimates of item parameters between groups after controlling the construct being measured. Uniform DIF exists when the b parameters are statistically different. With a non-uniform DIF, the discrimination parameters are significantly different across groups (DeMarc, 2010; Embretson et al., 2000; Ostini, 2006). Detecting DIF through GRM requires a two-stage process (Orlando Edelen, Thissen, Teresi, Kleinman, & Ocepek-Welikson, 2006). In the first step, which is an iterative procedure, anchor items (items without DIF) and study items (items with DIF) will be identified by comparing a compact model with an augmented model. In the first iteration and for each item, a model in which all parameter estimates are constrained to be equal for the child and parent groups (compact model) is compared with a model in which the parameters for the studied item are free to be estimated distinctly for the two groups (augmented model). The difference in the -2 log Likelihood of these models distributes as chi-square with m degrees of freedom (i.e., m-1 degrees of freedom when the thresholds are estimated and 1 degree of freedom when the slope is estimated) (Langer, 2008; Orlando Edelen, 2006). For each item, the significance of this value is considered an indication of DIF. After identifying temporary anchor items, items displaying DIF are eliminated from the anchor and the process is repeated until no items are identified as containing DIF. After finding a common set of items that contain no DIF, in the second stage, each of the studied items is reassessed for DIF with the use of a purified anchor set. It is likely that some items identified as having DIF in previous stages of the analyses, convert to non-DIF relative to the anchor items.

If an item shows significant DIF, the follow-up tests will be performed to detect which type of DIF (uniform or non-uniform) is displayed. The Benjamini-Hochberg (BH) procedure has been used for controlling the false discovery rate (FDR). If P_(1)_ < P_(2)_ < … P_(i)_ < … < P_(K)_ are the ordered P-values for K study items, in the BH method, each P_(i)_ will be compared with 0.05i/K (Benjamini & Hochberg, 1995; Williams, Jones, & Tukey, 1999). This study used IRTPRO2.1 to detect uniform and non-uniform DIF. IRTPRO uses Bock and Aitkin's marginal maximum likelihood (MML) estimation method for fitting models and estimating parameters as the default estimation algorithm (IRTPRO, 2011). Item information functions and item expected score curves were used to assess the effect and magnitude of DIF on the items and the subscales. The item expected score curve is a function of θ and provides better understanding of the uniform and non-uniform DIF across children and parents. Moreover, item information is a function of θ and provides valuable insight into the precision of subscale provided by the item (IRTPRO, 2011).

## 3. Results

**Figure 1:**
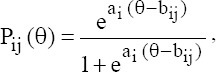
Expected item score function of DIF items for children (solid line) and parents (dashed line)

Table 1 shows the estimated parameters for the final anchor set, which are equal for both children and parents. In this table, item parameter estimates for the social subscale are not presented. This is because in the first step of the DIF analysis we did not find any items with DIF across children and parents in the social subscale. Table 2 presents the estimation of discrimination and threshold parameters for each study item separately for children and their parents. The uniform and non-uniform DIF tests are reported in the two last columns of the table. Using the B-H adjustment, 8 out of 23 items were identified as showing DIF. We found that 50% (4 out of 8) of the items in the physical subscales and 40% (2 out of 5) in both emotional and school subscales were flagged with DIF. Among the DIF items, 62.5% (5 out of 8) were uniform and the remaining 37.5% (3 out of 8) were non-uniform. The two DIF items in the emotional subscale were uniform, whereas two items in the physical functioning were uniform and the other two items operated as non-uniform DIF. Moreover, one item in the school subscale showed uniform DIF and the other exhibited non-uniform DIF. As shown in Table 2, for item 4 in the physical functioning, item 5 in the emotional functioning and item 4 in the school functioning, threshold parameters for parents are shifted to the left compared with those for children, indicating that parents tend to score in higher categories on those items (i.e., “never a problem” or “almost never a problem”). These results are better presented graphically in Figure 1.

**Table 1:** Item parameters and standard errors for anchor items used in the analysis of differential item functioning on the PedsQL™ 4.0 for children and parents

Items and domains	Group	a (S.E)	b_1_(S.E)	b_2_(S.E)	b_3_(S.E)	b_4_(S.E)
**Physical health**						
2. Hard to run	Child	2.18 (0.15)	-2.42 (0.13)	-1.65 (0.09)	-0.70 (0.05)	-0.06 (0.04)
	Parent					
3. Hard to do sports or exercises	Child	2.18 (0.15)	-2.46 (0.13)	-1.84 (0.10)	-1.04 (0.06)	-0.41 (0.04)
	Parent					
6. Hard to do chores around house	Child	0.79 (0.06)	-3.54 (0.25)	-2.48 (0.18)	-0.94 (0.09)	0.27 (0.08)
	Parent					
7. Hurt or ache	Child	0.87 (0.07)	-4.72 (0.35)	-3.21 (0.23)	-1.20 (0.09)	-0.07 (0.07)
	Parent					

**Emotional functioning**						
1. Feel afraid or scared	Child	1.26 (0.08)	-3.22 (0.20)	-2.38 (0.14)	-0.78 (0.07)	0.21 (0.05)
	Parent					
2. Feel sad or blue	Child	2.28 (0.15)	-2.17 (0.11)	-1.56 (0.08)	-0.43 (0.05)	0.33 (0.05)
	Parent					
3. Feel angry	Child	1.82 (0.11)	-1.98 (0.11)	-1.12 (0.07)	-0.00 (0.05)	0.80 (0.06)
	Parent					

**School functioning**						
1. Hard to concentrate	Child	1.59 (0.11)	-2.34 (0.14)	-1.67 (0.10)	-0.63 (0.05)	0.26 (0.06)
	Parent					
3. Trouble keeping up with schoolwork	Child	1.85 (0.13)	-2.00 (0.11)	-1.45 (0.08)	-0.56 (0.05)	0.19 (0.05)
	Parent					
5. Miss school – doctor appointment	Child	0.91 (0.09)	-3.95 (0.34)	-3.17 (0.27)	-1.62 (0.14)	-0.50 (0.07)
	Parent					

a: discrimination coefficient, b_i_: threshold parameters, S.E: standard error 0 = Almost always, 1 = Often, 2 = Sometimes, 3 = Almost never, 4 = Never

**Table 2:** Item parameters and standard errors for study items used in the analysis of differential item functioning on the PedsQL™ 4.0 for children and parents

							Test for DIF: χ^2^ (P)	
	Group	a(S.E)	b_1_(S.E)	b_2_(S.E)	b_3_(S.E)	b_4_(S.E)	a DIF	b DIF
**Physical health**								
1. Hard to walk more than a block	Child Parent	1.44 (0.13) 2.33 (0.23)	-3.29 (0.27) -2.44 (0.16)	-2.39 (0.18) -1.93 (0.13)	-1.23 (0.10) -1.12 (0.08)	-0.67 (0.07) -0.57 (0.06)	**11.5 (0.0007)**	7.20 (0.12)
4. Hard to lift something heavy	Child Parent	0.90 (0.09) 0.88 (0.09)	-3.02 (0.28) -3.72 (0.33)	-1.85 (0.17) -2.55 (0.22)	-0.08 (0.08) -0.76 (0.10)	1.06 (0.12) 0.51 (0.12)	0.00 (0.88)	**32.9 (0.0001)**
5. Hard to take a bath or shower	Child Parent	0.86 (0.14) 1.22 (0.13)	-4.19 (0.60) -1.86 (0.15)	-3.87 (0.55) -1.75 (0.14)	-3.32 (0.46) -1.44 (0.11)	-2.82 (0.38) -1.12 (0.09)	3.6 (0.06)	**84.6 (0.0001)**
8. Low energy	Child Parent	1.15 (0.11) 0.72 (0.08)	-3.79 (0.33) -6.39 (0.70)	-2.54 (0.21) -3.67 (0.36)	-1.28 (0.11) -1.82 (0.18)	-0.25 (0.07) -0.42 (0.11)	**11.9 (0.0005)**	5.20 (0.26)

**Emotional functioning**								
4. Trouble sleeping	Child Parent	1.14 (0.10) 1.26 (0.12)	-2.48 (0.20) -2.42 (0.21)	-1.80 (0.15) -1.75 (0.16)	-1.02 (0.10) -0.76 (0.09)	-0.22 (0.07) 0.02 (0.07)	0.60 (0.44)	**11.60 (0.02)**
5. Worry about what will happen	Child Parent	1.15 (0.10) 1.32 (0.12)	-1.65 (0.13) -1.88 (0.16)	-0.94 (0.09) -1.03 (0.10)	0.20 (0.07) -0.05 (0.07)	0.93 (0.10) 0.67 (0.09)	1.20 (0.27)	**12.50 (0.014)**

**School functioning**								
2. Forget things	Child Parent	1.44 (0.12) 0.97 (0.11)	-2.82 (0.21) -3.91 (0.38)	-1.90 (0.13) -2.57 (0.24)	-0.45 (0.07) -0.78 (0.10)	0.66 (0.07) 0.63 (0.13)	**8.20 (0.0041)**	6.5 (0.1635)
4. Miss school – not well	Child Parent	1.11 (0.12) 0.92 (0.13)	-3.59 (0.35) -4.39 (0.50)	-2.72 (0.25) -3.63 (0.40)	-1.44 (0.14) -2.41 (0.26)	-0.45 (0.08) -1.13 (0.13)	1.10 (0.2860)	**33.6 (0.0001)**

0 = Almost always, 1 = Often, 2 = Sometimes, 3 = Almost never, 4 = Never DIF: differential item functioning, a: discrimination coefficient, b_i_: threshold parameters, χ^2^: chi-square, p: p-value, S.E: standard error GJHS-5-89

The figure shows that, for item 4 of the physical subscale, item 5 of the emotional subscale, and item 4 of the school subscale, the expected score is higher for parents than for children. On the other hand, item 5 in the physical subscale and item 4 in the emotional subscale have the reverse pattern. For these items children tend to score in higher categories. That is, when the parents of children and the children themselves rate the HRQoL equivalently, children are more likely than parents to choose the higher response category. These patterns are best represented graphically in Figure 1.

**Figure 2:**
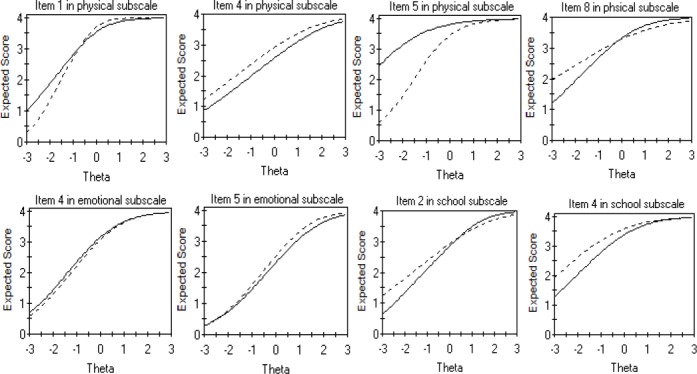
Item information function of DIF items for children (solid line) and parents (dashed line)

**Figure 3:**
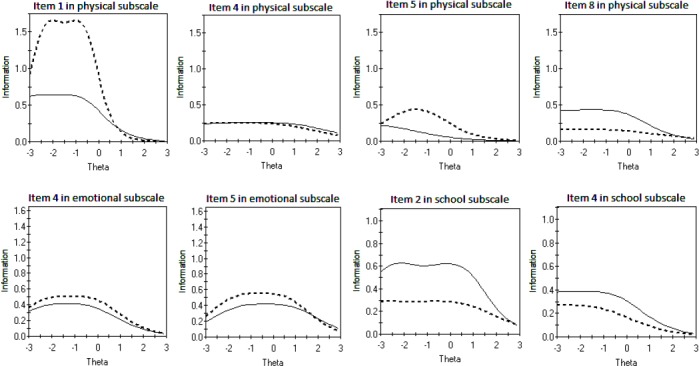
Total expected score for all eight items of the physical subscale, five items of the emotional subscale and five items of the school subscale for children (solid line) and parents (dashed line)

Figure 2 shows the item information function of DIF items in both groups. As compared with parents, items 2 and 4 in the children group provide more information about the school subscale. In contrast, items 4 and 5 in the parents group give more information about the emotional subscale than the children group. Moreover, compared to children, parents provide more information on items 1 and 5 in the physical subscale but less on item 8. By comparing results from Table 2 and Figure 2, we notice that items with higher discrimination indices give more information. Figure 3 displays the total expected score for all eight items of the physical subscale, five items of the emotional subscale and five items of the school functioning subscale for children and parents. The total expected score does not differ for children compared with parents across the range of emotional function. According to the parents, almost 90% of children did not have a chronic condition, indicating thatthe majority of children who participated in this study were apparently healthy.

## 4. Discussion

This manuscript investigates whether parents and children perceive the meaning of items in the PedsQL™ 4.0 differently. It examines this phenomenon based on IRT and using a powerful model (GRM) which has never been used by extant studies regarding child-parent agreement. This study revealed that parents and children respond differently to 8 out of 24 items in the PedsQL™ 4.0 questionnaire. However, a question that arises is whether the differences are substantial enough so that we should change the way in which this instrument is used or interpreted. This issue depends on the type and magnitude of DIF. The middle panel in Figure 3 shows that the total expected score dose not differ between children and parents for the five items on the emotional subscale overall. This is because, not only was the discrepancy in the item expected score negligible across children and parents on items 4 and 5, but these items with uniform DIF are in opposite directions. Hence, the HRQoL scores can be compared across groups in the emotional subscale as well as social subscale. These findings are in line with previous studies, which reported higher parent–child agreement for the social and emotional subscales (Eiser, Vance, Horn, Glaser, & Galvin, 2003; Varni & Burwinkle, 2006). In contrast, the slight difference in the total expected score provided by the physical and school subscales are practically important. A possible explanation is that the difference in the item expected score and/or item information function was considerable between children and parents on all DIF items in the physical and the school subscales. This finding does not support the hypothesis in other PedsQL™ 4.0 publications that more observable domains such as the physical functioning would yield higher agreement among children and their parents (Felder & Frey, 2004; Varni, Burwinkle, Katz, Meeske, & Dickinson, 2002; Varni, Burwinkle, Rapoff, Kamps, & Olson, 2004; Uzark, Jones, Burwinkle, & Varni, 2003). These results highlight to what extent agreement at the item level can be different from that at the scale level. In general, the total expected score in Figure 3 revealed that children were less optimistic about their school functioning and a little more optimistic about their physical functioning than their parents. On the other hand, they were in close agreement on aspects of social and the emotional functioning. Moreover, certain items in the anchor sets (Table 1) and most of the study items (Table 2) have quite low discrimination coefficients (a-values), indicating that these items do not provide as much information about the construct they belong to. Also, according to b-values in Tables 1 and 2, all items seem to be on the “easy” side, which provides sufficient evidence to confirm that the majority of children who participated in this study are healthy.

Finally, our findings were different from those of the two previous studies in the U.S. and China, which evaluated the measurement equivalence of the PedsQL™ 4.0 across child self-reports and parent proxy-reports. Interestingly, although our sample was similar to those used in China, the items did not function in the same way across the two populations. It is not clear whether the discrepancy is due to cross-cultural differences or the statistical testing methods. One possible explanation for differences between countries is that the words and phrases in the translated versions may not convey similar meanings and ideas to the source version. Moreover, it can be attributed to the children's social desirability as the tendency of children to manage social interactions by projecting favorable images of themselves (Johnson & Van de Vijver, 2003). While the Chinese version of the PedsQoL™ 4.0 confirmed interchangeability between child self-reports and parent proxy-reports in healthy children (Huang et al., 2009), the American version in children with chronic conditions showed that some of the items were flagged with DIF across the groups (items 2, 4, 5 and 6 in the physical subscale, item 1 in the emotional subscale, and items 1 and 5 in the social subscale) (Lin et al., 2012). While the IRT method used in the present study is designed for polytomous test items, the two previous studies were done with ordinary linear MCFA, which assumed that observed items were continuous and normally distributed (Meade & Lautenschlager, 2004). Hence, it cannot be justified to compare our findings with those of the two previous studies. Unlike MCFA, multi-group categorical confirmatory factor analysis (MCCFA) can appropriately model the ordered-categorical measures, and, accordingly, it is better comparable to the corresponding analytic technique in IRT (Kim & Yoon, 2011). As shown by Kankaras and colleagues, scalar and metric inequivalence in MCCFA is conceptually similar to uniform and non-uniform DIF in IRT model (Kankaras, Vermunt, & Moors, 2011).

The major limitation of our study is that it does not allow the researcher to adjust the items with DIF due to additional variables (e.g., child and parent sex, child's age, and child's health status) in the model. Hence, to determine how far these covariates contribute to the observed discrepancies between children and their parents, analysis of DIF according to these factors is also needed. A reasonable conclusion that can be drawn from our findings and those of the previous studies using the PedsQL™ 4.0 is that detecting DIF across child self-reports and parent proxy-reports in the PedsQL™ 4.0 depends mainly on the child health status, cross-cultural differences and, of course, on the statistical method used to explore DIF. We highly recommend and advocate the use of various statistical methods in DIF analysis because convergent findings through different methods can help researchers to remove or modify items with consistent DIF (Yang & Heslin, 2011).

## 5. Conclusion

In conclusion, our study revealed that in 3 of the 4 subscales, including the physical, emotional and school functioning, some items in the Persian version of the PedsQL™ 4.0 did not function in a similar way across children and their parents. Therefore, professionals and clinicians should be cautious about using parent proxy-reports as a substitute for children's own ratings. Future studies should consider comparing child self-reports and parent proxy-reports across different pediatric quality of life measures.
